# The value of grip test, lysophosphatidlycholines, glycerophosphocholine, ornithine, glucuronic acid decrement in assessment of nutritional and metabolic characteristics in hepatitis B cirrhosis

**DOI:** 10.1371/journal.pone.0175165

**Published:** 2017-04-06

**Authors:** Qing Ye, Weili Yin, Lei Zhang, Huijuan Xiao, Yumei Qi, Shuye Liu, Baoxin Qian, Fengmei Wang, Tao Han

**Affiliations:** 1 The Third Central Clinical College of Tianjin Medical University, Tianjin, PR China; 2 Department of Gastroenterology and Hepatology, Tianjin Key Laboratory of Artificial Cells, Tianjin Institute of Hepatobiliary Disease, Tianjin Third Central Hospital, Tianjin, PR China; 3 Clinical Laboratory of Tianjin Third Central Hospital, Tianjin, China; 4 Department of Nutriology, Tianjin Third Central Hospital, Tianjin, China; Taipei Veterans General Hospital, TAIWAN

## Abstract

The liver is essential for the regulation of energy, protein and amino acids, as well as in other aspects of metabolism. To identify efficient indexes for evaluation of nutritional status and metabolic characteristics during different Child-Pugh stages of hepatitis B cirrhosis, 83 patients and 35 healthy individuals were enrolled in our study. We found that grip strength, triceps skinfold thickness (TSF), body fat and skeletal muscle of the patients were reduced compared to the control group (*P*<0.05). Ultra-high-performance liquid chromatography data combined with mass spectrometry (UPLC-MS) showed that levels of a variety of metabolites, including lysophosphatidylcholines (LysoPCs), glycerophosphocholine, ornithine and glucuronic acid were reduced in the serum of patients with hepatitis B cirrhosis (*P*<0.001). However, glycerophosphoserine and taurocholic acid levels were higher than in the control group (*P*<0.001). Moreover, grip strength was correlated with the Child-Pugh score (*P*<0.05). Serum albumin, total cholesterol, LDL, LysoPCs, glycerophosphocholine, ornithine, glucuronic acid, glycerophosphoserine and taurocholic acid were correlated with the Child-Pugh score (*P*<0.01). These findings suggested that grip strength and the above small molecular substances might be considered as sensitive and important indexes for evaluating nutritional status and metabolic characteristics of patients with hepatitis B cirrhosis, which may help assess prognosis and adjust nutritional treatment.

## Introduction

The liver is the central organ for metabolism in the human body. When cirrhosis develops, liver structure abnormalities occur, liver functions change and barriers develop within metabolic pathways. Almost all cirrhosis patients have varying degrees of malnutrition and metabolic disturbances [1–3]. Studies have indicated that the incidence and severity of malnutrition are associated with the liver disease process and that malnutrition affects patient prognosis [2, 3]. Therefore, the accurate evaluation of nutritional and metabolic characteristics in patients with cirrhosis has significant meaning, which can provide a basis for nutritional therapy.

In this study, nutritional and metabolic features of patients with hepatitis B cirrhosis were assessed synthetically through the comparison of changes in anthropometry, body composition and serum metabolic indexes. Anthropometric indices including grip strength, triceps skinfold thickness (TSF), mid-upper arm muscle circumference (MAMC) and body mass index (BMI). Human bioelectrical impedance analysis (BIA) is a method of evaluating body composition. Biomedical information related to human physiology and pathology can be extracted by using electrical characteristics and the activities of biological tissues and organs. Body fat, protein content and skeletal muscle can be analyzed by studying the variation of the resistance of the same electrode under different frequencies. The objectivity and accuracy of this process in most cases have been affirmed. Although it still has some limitations, human body composition analysis has been applied to many areas of disease, such as chronic hepatitis, kidney disease, malnutrition, malignant tumors [4–7].

Additionally, the ultra-high-performance liquid phase chromatography combined with mass spectrometry (UPLC-MS) research platform for metabolism has developed rapidly in recent years. It is capable of quantitative determination of the overall status and assessment of the direction of endogenous biological metabolism [8–10]. It can filter out the characteristic metabolites in liver-related diseases to evaluate patient condition and assess prognosis [10–12].

There has been a lack of clinically relevant medical data regarding the combination of anthropometry, BIA and UPLC-MS methods to assess the nutritional status and metabolic characteristics of patients with cirrhosis. Thus, the nutritional status of 83 patients with hepatitis B cirrhosis and 35 patients with normal physical examinations in Tianjin Third Central Hospital was investigated in this study. The nutritional status and metabolic characteristics of patients with cirrhosis were assessed by the application of anthropometry, BIA and metabonomics platform. The metabolic characteristics of the patients were further analyzed to explore the potential clinical value and to seek new targets for nutritional treatment.

## Method

### Patients

Eighty-three patients with hepatitis B cirrhosis admitted to Tianjin Third Central Hospital between August 2014 and June 2015 were selected as the case group, including 42 males and 41 females, ages 38 to 65 years old, with an average of 54.4 years of age. Thirty-five individuals with normal physical examinations comprised the control group, including 20 males and 15 females, ages 35 to 64 years old, with an average of 54.0 years of age. All participant enrolled in this study ate a regular diet and denied any history of drinking. Diagnostic criteria for cirrhosis were used in accordance with the "prevention and treatment of viral hepatitis" [13] guidelines established in 2000. Excluded criteria were (1) patients with malignant tumors; (2) patients with hepatic coma; (3) patients with heart failure and renal insufficiency; (4) patients with diabetes, thyroid disease or other metabolic-related diseases; (5) patients with limb movement disorders; (6) patients who could not cooperate with the measurement; (7) patients who were fasting for more than one day; (8) patients with moderate or higher-grade anemia; and (9) patients who did not agree to participate in the study. This study was approved by the ethics committee of Tianjin Third Central Hospital. All clinical data were collected with the written informed consent of patients and members of the healthy control group. The relative ethics audit information is shown in S1 Table.

### Serum biochemical indexes

Serum albumin, triglyceride, total cholesterol, high density lipoprotein (HDL), low density lipoprotein (LDL), fasting blood glucose (FBS) of the patients and the healthy volunteers were tested using Japan Toshiba TBA-2000FR automatic biochemical analyzer at early morning in the fasting state.

### Anthropometry indexes

Height, body weight, grip strength (dominant hand), triceps skinfold thickness (TSF) and mid-upper arm muscle circumference (MAMC) were measured by two trained physicians (Qing Ye, attending physician in liver disease, with 10 years of clinical experience; and Hui-juan Xiao, a nutritional practitioner, with seven years of clinical experience) together in the next morning after hospitalization.

### Multi-frequency bioelectrical impedance (BIA)

The Biospace In Body3.0 human body composition analyzer (Biospace Company, Korea) was used to measure body fat, protein content and skeletal muscle. Electronic and metal goods were removed when each test subject was measured in morning fasting state with bowels relieved. The electrode was wiped with an alcohol-treated cotton ball. The test subject lay on the test bed, barefoot with arms abducted 15 degrees. The electrode was then connected, and previously collected basic information (age, gender, height) was entered until the machine displayed body mass. After 1 to 2 minutes, the measurement was conducted. BIA was performed by the whole body tetrapolar contact electrode approach using a multifrequency impedance analyzer applying alternating electric currents of 100 mA at 1 kHz, and of 800 mA at 5, 50, and 100 kHz.

### Equipment and reagent kits

The ultra-high-performance liquid chromatography and the LTQ Orbitrap XL mass spectrometry instruments were purchased from Thermo Fisher Scientific (Waltham, MA, USA). Acetonitrile (chromatographic purity) and formic acid (chromatographic purity) were purchased from Merck (Germany). The standards including lysoPC (16:0), lysoPC (17:0), lysoPC (18:0) and taurocholate were purchased from Sigma-Aldrich (USA). Distilled water was filtered by the Milli-Q system (Millipore, USA) before use.

### Ultra-high-performance liquid chromatography conditions

The serum sample was thawed, and 100 μL was collected. This aliquot was mixed with 300 μL acetonitrile to form a volume ratio of 1:3. After vigorous shaking for 30 seconds, followed by centrifugation at 12,000 g for 5 minutes at 4°C, the supernatant was removed and was filtered by a 0.22-μm filter (Millipore) to obtain the sample to be tested. Liquid chromatography was performed using the Accela ultra-high-performance liquid chromatography system from Thermo Fisher Scientific. The chromatographic column was a Thermo Hypersil GOLD reversed phase C18 column (2.1 mm ID×50 mm, 1.9 μm). The two-solvent gradient chromatography elution mode was used. Mobile phase A was 0.1% formic acid aqueous solution. Mobile phase B was 0.1% formic acid acetonitrile solution. The chromatographic elution process was 15 min, the injection volume was 10 μL, and the flow rate was set to 200 μL/min. The auto-sampler temperature was set to 4°C, and the column temperature was set to 20°C. The initial gradient was 5% B and was maintained for 2.5 min. At 8.5 min, the gradient increased to 95%. It was later decreased to 5% B after 3 min of maintenance. After that, 2.5 min was taken to balance the chromatography column.

### Mass spectrometry conditions

Mass spectrometry was performed using the LTQ Orbitrap XL system from Thermo Fisher Scientific, equipped with an electrospray ion source (ESI) and positive ion mode for detection. The conditions were as follows: Capillary voltage was 7 v, cone voltage was 50 v, ion source voltage was 4.8 kV, data acquisition range was m/z 50–1,000, rod scan mode was used, and the resolution was 100,000. Mass axis correction used the correction fluid provided by the manufacturer (including caffeine, Ultramark1621 and tetrapeptide MRFA). Excel's randomization function generated the sequence of sample analysis. The UPLC-MS research platform was analyzed to obtain data. The data were then imported into MZ mine 2.0 software for pre-data processing [14]. According to the rule of extracted ion chromatographic peak signal-to-noise ratio > 30, the retention time offset did not exceed 0.1 min and the mass charge ratio deviation did not exceed ±0.01 during peak recognition, matching and normalization processing. The data were imported into SIMA-P+12.0.1.0 (Umetrics, Sweden) for analysis. Principal component analysis (PCA) and orthogonal partial least squares discriminant analysis (OPLS-DA) modes were constructed by the pattern recognition method [15]. The potential markers were selected according to the contribution degree of the electrospray ion for the mode and confidence interval.

### Identification of metabolic markers

Some of the characteristic ions were identified by comparison between the standard spectral peaks and peaks of the mass spectra. There was no standard to compare the characteristics of the ion identification method. (1) Due to the first order mass spectrometry scan using Orbitrap XL mass spectrometry and its resolution being 100,000 (FMHW), the accurate mass charge ratio m/z value of ion characteristics was used for searching the HMDB (http://hmdb.ca/) database. According to the rule of deviation, the m/z value could not exceed 0.02, the exact number of charges, and the ionization mode had to be consistent with experimental conditions. The retrieval results were checked, and the material identification that matched the requirements was retained. (2) The characteristic ions were scanned by MS/MS, and their secondary mass spectra were obtained. The spectra were compared with the theoretical fragments of HMDB identification results simulated by the Mass Frontier 6.0 software database (Thermo Fisher Scientific). Finally, it was ensured that the content of phospholipids, long chain acyl carnitine and adenosine had significant differences according to the following principles. First, the comparison deviation between secondary mass spectrum m/z values of characteristics of ions and theory fragmentation was less than 0.2 (secondary mass spectrum fragmentation was generated by ion trap mass spectrometry CID mode). Second, theory fragmentation could match the three peaks of two pieces of characteristic ions as well as cover the mass spectrum of secondary fragmentation of the characteristic ions by more than 80%. Finally, there had to be a significant difference in the content of the three types of substances, such as phospholipids, long chain acylcarnitine and adenosine.

### Statistical analyses

SPSS v16.0 software (SPSS Incorporated, Chicago, Illinois, USA) was used. The normality and homogeneity of variance of the indexes of each group were tested. For the indexes according to normal distribution, single-factor variance analysis was conducted. If the variances satisfied the homogeneity, multiple comparisons were conducted using the LSD method. If the variances did not satisfy the homogeneity, multiple comparisons were performed using Dunnett's T3 method. The means and standard deviation (SD) were used for expression of indices with normal distributions. The median was used for the representation of indices that did not conform to a normal distribution. For indices that did not conform to the normal distribution, K-W non-parametric tests were performed. Multivariate statistical analysis was conducted using SIMCA-P. The orthogonal partial least-squares discriminant analysis (OPLS-DA) model was constructed through the SIMCA-P+12.0.1.0 pattern recognition method. The potential markers were preliminarily screened according to the VIP values and VIP confidence intervals. Values with *P*<0.05 had statistical significance.

## Results

### Biochemical indicators changes in hepatitis B cirrhosis patients

Albumin, triglyceride, total cholesterol, HDL, LDL and FBS were determined by automatic biochemical analyzer (Table 1). The level of albumin, total cholesterol, HDL and LDL decreased significantly, along with the decrement of liver compensatory ability (F = 25.53, *P*<0.001; F = 9.88, *P*<0.001; F = 5.22, *P* = 0.007; F = 11.37, *P*<0.001, respectively).

**Table 1:** Serum biochemical indexes in the case groups and control group.

	Child-Pugh A group	Child-Pugh B group	Child-Pugh C group	Control group	*P* value
	N = 33	N = 29	N = 21	N = 35	
**Age (year)**	53.82±8.12	52.93±9.24	57.19±10.04	54.03±7.53	0.361
**Male/female (%)**	48.5/51.5	51.7/48.3	52.4/47.6	57.1/42.9	0.914
**Albumin (g/l)**	34.72±1.81	30.46±4.72*	25.33±3.89&*	41.40±2.30*&#	<0.001
**Triglyceride (mmol/l)**	1.09±0.32	1.71±0.38	0.87±0.24	1.36±0.26	0.349
**Total cholesterol (mmol/l)**	4.45±1.02	3.25±1.32*	2.27±0.53*	4.91±0.26&#	<0.001
**HDL (mmol/l)**	1.37±0.22	0.68±0.16*	0.72±0.12*	1.28±0.11&#	0.007
**LDL (mmol/l)**	2.75±0.52	1.95±0.66*	1.17±0.11&*	2.63±0.42&#	<0.001
**FBS (mmol/l)**	5.02±0.77	4.96±1.28	4.65±1.16	5.56±1.87	0.529

Notes: Means ± SD. Single-factor variance analysis was conducted after verifying the data all accord with the normal distribution in this table.

HDL: High density lipoprotein; LDL: Low density lipoprotein; FBS: Fasting blood glucose.

*Compared with Child-Pugh A group, P<0.01

&compared with Child-Pugh B group, P<0.01

#compared with Child-Pugh C group, P<0.01.

### Grip strength decreased in hepatitis B cirrhosis patients

The nutritional status of patients with hepatitis B cirrhosis was assessed by anthropometric indexes (Table 2). Grip test measured hand and forearm muscle strength. It could reflect the function of skeletal muscle and is analyzed through a method that is easy to perform and may provide a very sensitive index for assessment of nutritional status and hepatic compensatory ability [16]. In comparing Child-Pugh A, B and C groups with the normal control, it was found that, along with reduced liver compensatory capacity, grip strength decreased significantly (F_totle_ = 15.766, *P*<0.001; F_male_ = 25.392, *P*<0.001; F_female_ = 27.106, *P*<0.001). The grip strength of patients, even those in Child-Pugh A, decreased significantly compared to the control group once the disease progressed to cirrhosis. This finding suggested that low muscle function had already occurred during the early stage of cirrhosis.

**Table 2:** Indexes of anthropometry and body composition analysis in the case groups and control group.

	totle					male					female				
	Child-Pugh A group	Child-Pugh B group	Child-Pugh C group	Control group	*P* value	Child-Pugh A group	Child-Pugh B group	Child-Pugh C group	Control group	*P* value	Child-Pugh A group	Child-Pugh B group	Child-Pugh C group	Control group	*P* value
	N = 33	N = 29	N = 21	N = 35		N = 18	N = 13	N = 11	N = 20		N = 15	N = 16	N = 10	N = 15	
**Grip strength (kg)**	25.86±9.39	23.36±8.54	19.78±9.57*	36.27±11.11*&#	<0.001	33.39±7.87	27.01±6.52$	26.68±7.70$	43.79±8.99$%@	<0.001	19.83±6.10	17.51±4.11	12.45±4.70$&	26.94±4.64$%@	<0.001
**TSF (mm)**	19.72±4.44	17.16±6.82	15.10±7.05*	19.70±4.57#	0.009	20.31±5.19	17.67±6.12$	15.23±6.34$	20.39±3.27@	<0.001	20.08±4.17	16.49±3.16	16.28±5.21	22.29±5.38%@	<0.001
**MAMC (cm)**	22.20	20.00	20.90	21.70	0.153	22.37±2.12	22.22±3.05	22.15±3.16	22.65±1.89	0.904	22.03±2.48	20.45±3.12	20.42±1.90	22.19±1.38	0.229
**BMI (kg/m**^**2**^**)**	23.74±3.78	22.09±4.26	22.40±4.29	23.75±3.84	0.242	24.36±4.19	22.37±3.40	23.41±6.14	24.49±3.35	0.129	23.68±4.36	21.91±3.78	24.39±5.31	24.61±3.97	0.389
**Fat (kg)**	19.70±7.58	14.95±7.30*	15.74±5.52*	19.57±7.22&#	0.047	19.61±9.52	15.20±6.69$	15.88±5.18*	19.75±6.59&#	0.042	20.15±7.18	14.28±5.78*	17.34±6.02	22.32±7.72%	0.048
**Skeletal muscle (kg)**	25.74±5.33	25.16±5.48	23.65±5.09	26.96±5.87#	0.049	30.77±7.72	28.03±4.22	27.47±4.86*	31.04±3.33#	0.015	20.72±2.66	20.59±3.30	20.03±4.20	22.30±3.34	0.131
**Protein (kg)**	9.19±1.7	9.03±1.82	8.67±1.79	9.67±1.94	0.139	10.54±1.55	9.97±1.43	10.0±1.70	10.93±1.12	0.254	7.51±0.89	7.52±1.09	8.04±1.69	8.14±1.22	0.145

Notes: The means and standard deviation (SD) were used for expression of indices with normal distributions. The median was used for the representation of indices that did not conform to a normal distribution. For the indexes according to normal distribution, single-factor variance analysis was conducted. For indices that did not conform to the normal distribution, K-W non-parametric tests were performed.

TSF: thickness of skin-fold; MAMC: mid-upper arm muscle circumference; BMI: body mass index.

*Compared with Child-Pugh A group, P<0.05

&compared with Child-Pugh B group, P<0.05

#compared with Child-Pugh C group, P<0.05.

$Compared with Child-Pugh A group, P<0.01

%compared with Child-Pugh B group, P<0.01

@compared with Child-Pugh C group, P<0.01.

TSF can reflect the subcutaneous fat content, was decreased in hepatitis B cirrhosis male patients with Child-Pugh C group than the Child-Pugh A or control group (F_male_ = 6.056, *P*<0.001), while it decreased with Child-Pugh B and C groups than normal control in female subjects (F_female_ = 5.921, *P*<0.001). Nevertheless, BMI and MAMC did not show significant differences between groups (BMI: F_male_ = 1.216, *P* = 0.129, F_female_ = 1.416, *P* = 0.389; MAMC: F_male_ = 0.216, *P* = 0.904, F_female_ = 1.416, *P* = 0.229).

### Comparison of human body composition measured by BIA

Body fat, skeletal muscle and protein content are important indices to evaluate changes in body composition and were measured by using a human body composition analyzer to assess the changes (Table 2).

In male patients, Body fat content with Child-Pugh B and C group was significantly lower than with the Child-Pugh A and control group (F_male_ = 2.692, *P* = 0.042). In the Child-Pugh C group, skeletal muscle was significantly lower than in the Child-Pugh A and control group (F_male_ = 3.562, *P* = 0.015). However, the body protein level did not show statistical meaning (F_male_ = 1.35, *P* = 0.254).

In female patients, body fat content with Child-Pugh B group was significantly lower than with the Child-Pugh A and control group (F_female_ = 2.355, *P* = 0.048). There were no significant differences in skeletal muscle and protein between each group (Skeletal muscle: F_female_ = 2.172, *P* = 0.131; Protein content: F_female_ = 2.195, *P* = 0.145).

### Analysis of serum metabolic profile in patients with hepatitis B cirrhosis

**Fig 1:**
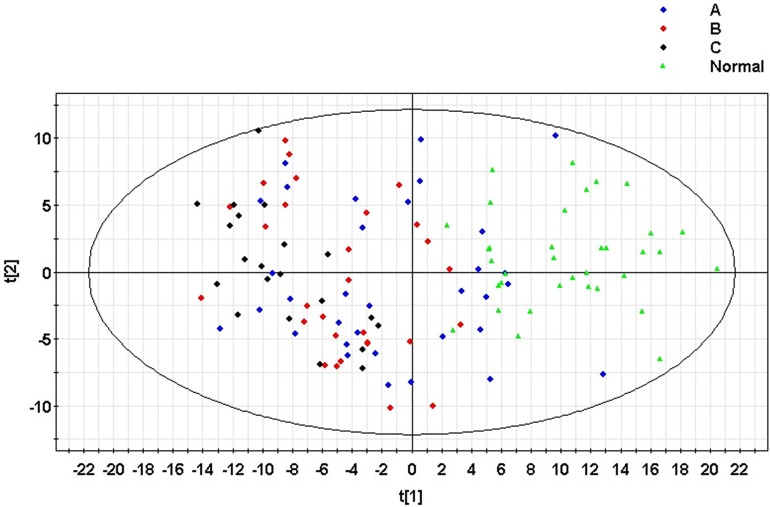
The principal component (PCA) model of the serum samples from patients with hepatitis B cirrhosis and normal controls. A: Child-Pugh A group; B: Child-Pugh B group; C: Child-Pugh C group. Normal: Control group.

**Fig 2:**
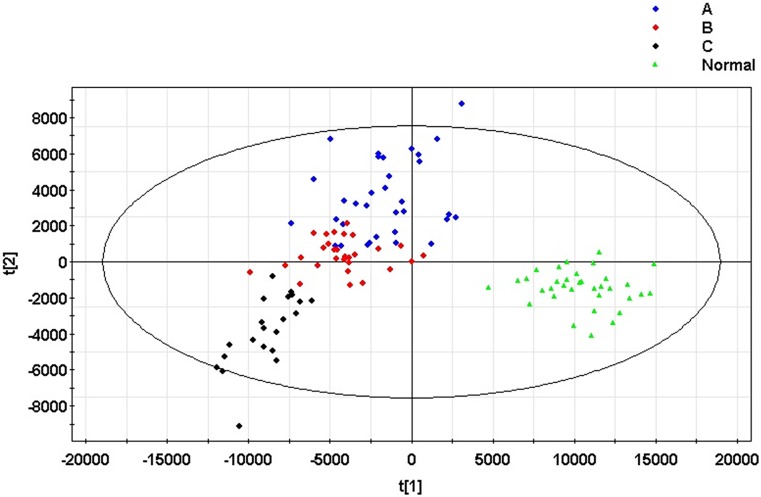
The orthogonal partial least squares discriminant analysis (OPLS-DA) model of patients with hepatitis B cirrhosis and the normal control group serum samples. A: Child-Pugh A group; B: Child-Pugh B group; C: Child-Pugh C group. Normal: Control group. In the second prediction of principal component orientation (Y axis), the serum metabolic profile of patients with different stages of cirrhosis of the liver can be distinguished by clustering. The second prediction of principal component orientation represents the development trend of hepatitis B cirrhosis.

A principal component analysis (PCA) model was established for all samples. The model parameters were R2X = 38.9% and Q2 = 27.3%. The scoring graph of the first principal component (t[1]) direction and the second principal component (t[2]) direction are shown in Fig 1. The model can distinguish clustering for the normal control group and patients with hepatitis B liver cirrhosis. At the same time, the model showed no outliers, indicating that the instrument was stable and the results were reliable. At the same time, all samples were constructed using orthogonal partial least squares discriminant analysis (OPLS-DA) models. The model parameters were R2X = 68.4%, R2Y = 76.1% and Q2 = 59.7%. The scoring graph of the first principal component (t[1]) direction and second principal component (t[2]) direction is shown in Fig 2. There was an obvious trend of clustering between groups.

### Changes of metabolic and nutritional characteristics in hepatitis B cirrhosis patients

Characteristic ions in the model were screened by the pattern recognition method. Through the comparison between two-stage mass spectrometry and the standard spectrum, 16 metabolites were identified (Table 3).

**Table 3:** Extraction ion chromatography peak integral area of the characteristic metabolites.

Identity	Child-Pugh A group N = 33	Child-Pugh B group N = 29	Child-Pugh C group N = 21	Control group	*P* value
				N = 35	
**Lysophosphatidylcholine (16:0)**	1530000.00	31460.00	6056.68*	4680000.00*&#	<0.001
**Lysophosphatidylcholine (17:0)**	322815.00	186535.00	2141.90*	809925.00*&#	<0.001
**Lysophosphatidylcholine (18:0)**	18800000.00	16000000.00	6700000.00*	30800000.00#	<0.001
**Lysophosphatidylcholine (18:1(11Z))**	10684655.28±6108967.75	5544368.8±1029563.45	5448814.36±1189028.77*&	30761741.89±635850.43*&#	<0.001
**Lysophosphatidylcholine (18:3(9Z,12Z,15Z))**	157973.00	129300.00	1250.86	411258.00*&#	<0.001
**Lysophosphatidylcholine (P-18:1(9Z))**	1020000.00	771513.00	400995.00*	2060000.00*&#	<0.001
**Lysophosphatidylcholine (20:1(11Z))**	55643.00	4249.10	864.05*&	4881.41#	<0.001
**Lysophosphatidylcholine (20:3(8Z,11Z,14Z))**	99741.47±812192.07	885900.16±698239.50	570971.71±708520.83*	1857051.58±803676.67*&#	<0.001
**Glycerophosphocholine**	579257.00	578396.00	342830.00	1230000.00*&#	<0.001
**Ornithine**	48179.60	45454.60	28218.10*	59165.10&#	<0.001
**Glucuronic acid**	2141.92	1535.52	1113.32	243681.00*&#	<0.001
**Glycerophosphoserine**	1353.22	3313.70*	16705.00*	1449.68&#	<0.001
**Taurocholic acid**	866.41	146417.00	86805.40	107.48*&#	<0.001
**D-glucose**	995051.00	1440000.00	1330000.00	982333.00	0.104
**Citrus flavin**	4427.99	4859.30	4940.02	5547.47	0.118
**Pregnanetriol**	11563.90	14285.80	14297.80	15339.30	0.147

Notes: The means and standard deviation (SD) were used for expression of indices with normal distributions. The median was used for the representation of indices that did not conform to a normal distribution. For the indexes according to normal distribution, single-factor variance analysis was conducted. For indices that did not conform to the normal distribution, K-W non-parametric tests were performed.

*Compared with Child-Pugh A group, P<0.05

&compared with Child-Pugh B group, P<0.05

#compared with Child-Pugh C group, P<0.05.

These metabolites were involved in various metabolic processes, including lipid metabolism (lysophosphatidylcholine and glycerophosphoserine), amino acid metabolism (Ornithine), bilirubin metabolism (glucuronic acid), bile acid metabolism (taurocholic acid), sugar metabolism (D-glucose) and hormone metabolism (pregnanetriol). Serum metabolites were compared among the patients with hepatitis B cirrhosis and the normal control (Table 3). Eleven substances decreased along with the apparent reduction of liver compensatory ability, include: lysoPCs (16:0, 17:0, 18:0, 18:1 (11Z), 18:3(9Z, 12Z, 15Z), P-18:1 (9Z), 20:1 (11Z), (20:3 (8Z, 11Z, 14Z)), glycerophosphocholine, ornithine, and glucuronic acid (*P*<0.001). The levels of two substances were increased with the reduction in liver compensatory ability, which were glycerophosphoserine and taurocholic acid (*P*<0.001). The results showed that the abnormal metabolism of liver cirrhosis affected almost all of the major biochemical processes of the human body.

### Correlation between nutritional metabolic indexes and Child-Pugh score

The relationship between nutritional and metabolic indices and Child-Pugh score was analyzed by using Pearson correlation analysis (Table 4). It is apparent that changes in albumin, total cholesterol, LDL, grip strength, lysoPCs (16:0, 17:0, 18:0, 18:1 (11Z), 18:3(9Z, 12Z, 15Z), P-18:1 (9Z), 20:1 (11Z), (20:3 (8Z, 11Z, 14Z)), glycerophosphocholine, ornithine, glucuronic acid, glycerophosphoserine and taurocholic acid were correlated with Child-Pugh score, suggesting that these indexes could be used as an indicator of the hepatic compensatory capacity.

**Table 4:** Correlation between each index and the Child-Pugh score.

Explanatory variable	Correlation coefficient	*P* value
**Albumin**	-.764**	.000
**Total cholesterol**	-.712**	.000
**LDL**	-.782**	.000
**Grip strength**	-.228*	.038
**Lysophosphatidyl choline (16:0)**	-.447**	.000
**Lysophosphatidyl choline (17:0)**	-.397**	.000
**Lysophosphatidyl choline (18:0)**	-.430**	.000
**Lysophosphatidyl choline (18:1(11Z))**	-.271*	.013
**Lysophosphatidyl choline (18:3(9Z,12Z,15Z))**	-.357**	.000
**Lysophosphatidyl choline (P-18:1(9Z))**	-.434**	.000
**Lysophosphatidyl choline (20:1(11Z))**	-.378**	.000
**Lysophosphatidyl choline (20:3(8Z,11Z,14Z))**	-.234*	.034
**Glycerophosphocholine**	.288**	.008
**Ornithine**	-.365**	.001
**Glucuronic acid**	.309**	.005
**Glycerophosphoserine**	-.291**	.008
**Taurocholic acid**	-.291**	.008

*Significant at 0.05 level

**significant at 0.01 level.

## Discussion

During recent years, increasing attention has been paid to cirrhosis patients with nutritional and metabolic challenges because malnutrition and metabolic barriers are independent predictors of mortality in patients with cirrhosis [17]. These deficiencies are closely related to complications of a period of decompensation, such as ascites, primary peritonitis, hepatic encephalopathy, hepatorenal syndrome and quality of life [18, 19]. Therefore, the accurate evaluation of nutritional and metabolic characteristics in patients with cirrhosis has significant meaning, which can provide the basis for nutritional therapy.

### 1. Blood lipid -TSF -body fat- lipid metabolism

Under normal circumstances, the liver is the primary site of lipid biosynthesis and storage [20]. Low lipid levels often reflect compensatory of liver and nutritional status [21]. Since the liver plays a central role in cholesterol metabolism, liver disease can impact cholesterol metabolism [22]. Conversely, our research suggested various changes in cholesterol metabolism (total cholesterol, LDL) can be indicators of hepatic dysfunction.

TSF was wildly used for reflecting the subcutaneous fat content. Considering the stability of TSF, it was a more reliable indicator compared with BMI in body fat validation [19, 23], The TSF of the Child-Pugh C group was significantly lower compared to the Child-Pugh A group and the control group. This finding could reflect the high fat consumption of decompensated patients with cirrhosis.

The body fat content measured by BIA method in Child-Pugh B and C group was significantly lower than that in A group and control group. However, in Child-Pugh C group, the fat content of male and female patients was higher than that of B group. This phenomenon may be related to ascites in patients with Child-Pugh C group, which could affect the accuracy of BIA [24].

Lysophosphatidylcholine (LysoPC) is a major bioactive plasma lipid in the normal human circulation where it is predominantly associated with albumin and lipoproteins [25]. The compounds are derived from phosphatidylcholine (PC), also called lecithin, mainly catalyzed by lecithin-cholesterol acyltransferase (LCAT) [26]. Within high-density lipoproteins (HDL), LCAT catalyzes the transacylation of the fatty acid residue of PC to free cholesterol, which subsequently results in the formation of LysoPC and cholesterol ester [27]. Eight LysoPCs of various lengths and glycerophosphocholine were decreased in patients’ serum metabolic profiling, along with the decrement of total cholesterol, LDL, HDL, TSF and body fat content. This finding indicated that the significant reduction of serous lipid substances of hepatitis B cirrhosis patients was consistent with the significant reduction of body fat content, analyzed either through the BIA method or the anthropometry. The results of our study showed that total cholesterol, LDL, eight lysoPCs and glycerophosphocholine were significantly lower in cirrhosis group, correlated with the Child-Pugh score (P<0.001), which suggesting that lipid metabolism was overall impaired in patients with hepatitis B cirrhosis. This indexes might be used as indicators to assess lipid metabolism status.

### 2. Grip strength- Skeletal muscle content

Grip test measured hand and forearm muscle strength. It could reflect the content and function of skeletal muscle and is analyzed through a method that is easy to perform and may provide a very sensitive index for assessment of nutritional status and hepatic compensatory ability [28]. In comparing cirrhosis patients with the normal control in this study, it was found that, along with reduced liver compensatory capacity, grip strength decreased significantly. From the data, the grip strength of patients, even those in Child-Pugh A, decreased significantly compared to the control group once the disease progressed to cirrhosis. This finding suggested that low muscle content and energy consumption had already occurred during the early stage of cirrhosis.

Some studies suggested that measuring reduced skeletal muscle produced higher sensitivity and specificity than other evaluation methods of nutritional status [16, 29, 30]. Furthermore, for patients with cirrhosis, reduced muscle could seriously affect the quality of life and increase the risk of death [31–33]. In our study, the content of skeletal muscle measured by BIA was significantly lower in Child-Pugh C group than that in control group, especially in male patients.

### 3. Amino acid metabolism

The liver is the primary site of synthesis and decomposition of amino acids. Amino acids are widely involved in the metabolism of body proteins, energy and other substances. In patients with liver cirrhosis, the synthesis of amino acids decreases and the decomposition increases. The increased decomposition can lead to the increment of blood ammonia significantly and may be related to hepatic coma [34, 35]. Ammonia and carbon dioxide can produce urea through ornithine, citrulline and arginine acid via the ornithine cycle. Our study showed the ornithine level of patients was significantly decreased, which suggest preventive supplement of ornithine might be benefit for cirrhosis patients.

### 4. Bilirubin metabolism

The metabolism of bilirubin is generated by the combination of non-conjugated bilirubin (also known as indirect bilirubin) and glucuronic acid. We found that glucuronic acid levels were significantly reduced in patients with cirrhosis, which directly affected the metabolism of bilirubin. This finding was consistent with the exacerbation of jaundice concurrent with the reduction of hepatic compensatory ability [36].

### 5. Bile acid metabolism

Only a small amount of bile acid occurs in the peripheral blood of the healthy population. With the injury to liver cells, the creatinine clearance rate of bile acid declines, resulting in an increase in serum bile acid. Thus, bile acid is considered to be a biomarker of hepatic injury [37]. Taurocholic acid is a product of the combination of taurine in liver and bile acid. The increase in the concentration of taurocholic acid in the serum of patients with hepatitis B cirrhosis may be related to the occurrence of cirrhosis of the liver. But, Our data showed serum taurocholic acid with Child Pugh C group was lower than B group. This interesting phenomenon might associated with the accumulation of toxic and even carcinogenic bile acid in liver that may be caused by the alteration of the bile acid transport pathway [38].

### 6. BMI and MAMC

Some patients with liver cirrhosis, especially patients with decompensated liver function resulting in edema, ascites, pleural effusion and other fluid-retention manifestations, may affect the reliability of some indicators, such as BMI. As shown in our results, BMI and MAMC did not have a significant changes among experimental groups. Therefore, it is not recommended to use BMI and MAMC to evaluate the nutritional status of patients with liver cirrhosis, especially in decompensated stage.

### 7. BIA

BIA is a precise and non-invasive technique that measures lean body mass and fat stores, but it is not accurate enough when patients retain fluid, because it assumes that the human body resembles a cylinder of constant hydration and invariably lean mass [24]. Thus, there are some theoretical restrictions on the use of BIA for individuals with abnormal body composition. For hepatic cirrhosis patient, the body composition results might be affected by edema or ascites. However, Some other researchers reported that, despite the limitations in patients with ascites, BIA was still a reliable bedside tool for the determination of body cell mass in cirrhotic patients with and without ascites, comparing with CT or MRI assessment of skeletal muscle and fat, the BIA technique was simpler, more economical and safer, thus providing a reliable, noninvasive evaluation tool for nutrition status of patients with cirrhosis [39]. In this study, we did not exclude patients with edema and ascites but performed a comprehensive assessment of nutritional status by different measurements and serum indexes, in the aim of figuring out whether BIA have practical application value in evaluating the body composition of cirrhosis patients with or without fluid retention. Since some studies showed that in patients with liver cirrhosis, changes in body composition could be related to the development of liver disease and the Child-Pugh score [40], we conducted correlation analysis between the nutritional parameters and Child-Pugh score. It was found that, body fat, skeletal muscle and protein content did show a “decreasing trend” along with the severity of liver decompensation, but did not have correlation with the Child-Pugh score. In the Child-Pugh C group, which was composed by cirrhosis patients all had ascites in our research, the measured fat content was higher than that of the B group, which was not consistent with the nutritional status of patients with liver cirrhosis. This phenomenon may be related to ascites in patients with Child-Pugh C group, as mentioned above. Taken together, BIA may be used to rough reflect the malnutrition status of cirrhotic patients in Child-Pugh A and B stages, but is not a good tool to precisely assess body composition of cirrhosis patients with body fluid retention, especially in Child-Pugh C stages.

In view of the limitations of BIA, the clinically established bioelectrical impedance parameter is the phase angle [41]. BIA is represented by the vector Z, which is a combination of the perpendicular vectors R and Xc. The vector Z has a module M, and the horizontal axis defines the PA [42]. It may reduce the errors and biases. So we will further evaluate it in our future studies.

### 8. Metabolic profile of serum

Further application of the metabolomics method was used to analyze the data obtained by using principal component analysis (PCA) and orthogonal partial least squares (OPLS) for pattern recognition with the normal healthy group. The results showed that the prediction and interpretation of the model were sound, as a total of 16 substances were identified. The UPLC-MS method is useful for the detection of abnormal metabolism in serum, and may provide a target for clinical nutritional therapy.

The patients with hepatitis B cirrhosis demonstrated signs of malnutrition. Cholesterol, LDL, grip strength, lysoPCs (16:0, 17:0, 18:0, 18:1 (11Z), 18:3(9Z, 12Z, 15Z), P-18:1 (9Z), 20:1 (11Z), (20:3 (8Z, 11Z, 14Z)), glycerophosphocholine, ornithine, glucuronic acid, glycerophosphoserine and taurocholic acid were sensitive markers in the assessment of nutritional status. The observed characteristics of the small molecular substances may provide a therapeutic target for further treatment. Advancing potential targets for the treatment of nutritional metabolism is necessary for early intervention in patients with liver cirrhosis to improve prognosis and quality of life.

In China, patients with cirrhosis mainly have hepatitis B cirrhosis [43]. All disease cases included in this study were patients with hepatitis B cirrhosis. The difference exists in the nutritional processes of hepatitis B cirrhosis compared to cirrhosis resulting from other etiologies, such as alcoholic, autoimmune and other types of cirrhosis. The nutritional processes involved with these other forms of cirrhosis need further exploration in future research. Additionally, this study was conducted with data from a single center. Future research studies will need to collaborate with other centers to expand the sample size. Expansion of the study population along with a broader definition of the cirrhosis types will reduce possible bias and provide direction for necessary nutritional and metabolic assessments and interventions for a wider range of patients with cirrhosis.

## Conclusions

In summary, total cholesterol, LDL, grip strength, lysoPCs, glycerophosphocholine, ornithine, glucuronic acid were reduced along with the decrement of liver compensatory ability in patients with hepatitis B cirrhosis, suggesting that these parameters may serve as sensitive indices for evaluating the nutritional status. The changes of these small molecular metabolic substances may reflect the metabolic disturbance and may be useful for the nutritional treatment of patients with hepatitis B cirrhosis.

## Supporting information

S1 Table Relative ethics audit information. (PDF)
