# Supplementary material for: The value of grip test, lysophosphatidlycholines, glycerophosphocholine, ornithine, glucuronic acid decrement in assessment of nutritional and metabolic characteristics in hepatitis B cirrhosis
Source: PLoS One. 2017 Apr 6;12(4):e0175165. doi: 10.1371/journal.pone.0175165 (PMC5383249; doi:10.1371/journal.pone.0175165)
Supplement: S1 Table — (PDF) [file pone.0175165.s001.pdf]

研究项目、论文发表快速伦理审查表

研究名称：乙肝肝硬化患者的营养状态及血清代谢组学的初步研究	
负责人：叶青	所在科室：消化肝病科
主要研究者：叶青、尹伟利、张磊、肖慧娟、韩涛、齐玉梅、刘树业、钱宝鑫、王凤梅	
本研究为：<br>☐ 单纯实验室研究<br>☐ 不涉及任何人或人体标本等的研究<br>☐ 其他（请说明）：	本论文为：<br>☐ 单纯实验室所发表的论文<br>☐ 不涉及任何人或人体标本等的研究所发表的论文<br>☑ 其他（请说明）：<br>本研究应用患者及健康体检者的身体测量及血液化验指标数据，及检验后废弃血标本的实验数据，所有数据及废弃血标本使用前与患者及体检者沟通，知情同意后收集。<br>本研究方案经过天津市第三中心医院伦理委员会批准。

本研究/论文符合快速报送伦理委员会审查案件，故提出申请。

负责人签字：叶青

以下由伦理委员会填写

初审结果：

☑ 同意

☐ 不同意

☐ 修改后同意

建议：

审核委员签名：[illegible] 16年 12月 29 日

主任委员签名：[illegible] 16年 12月 29 日

临床试验患者知情同意书

尊敬的患者：

您好！您将作为____肝硬化患者营养代谢分析____临床试验的一名受试者。为了确保本次试验是在充分保障您的权益的前提下进行，在您同意参加之前，您需要清楚知道以下相关信息：

一、研究内容和性质

本研究的主要内容是测量肝硬化患者的身高、体重、上臂肌围、握力、身体成分分析等指标。并应用患者住院期间废弃的血液标本进行血清学检测，评估其代谢轮廓（免费进行）。本研究为观察性研究，非干预性研究。

二、保密责任

本次试验所取得的结果与资料归临床观察项目的实施者及医疗机构所有并无偿使用，但您的合法权益不会因为本项研究而受到侵犯，您的个人资料由我院保密。我院伦理委员会、实施者可以查阅您的资料，但均不得对外披露其内容。研究结果将在不泄露您的身份的前提下因科学研究目的而发表。

如果您已充分理解并同意上述内容，请在本知情同意书下方签字确认。

研究者签名：[illegible]　　　　日期：　15年 1月12日

医生已充分向本人介绍了本验证的目的、方法等内容，也充分告知了本人享有的权利和应该履行的义务，并对本人询问的所有问题也给予了圆满的答复。本人自愿参加本次实验性临床医疗，并积极配合医生完成本项验证工作。

受试者签名：[illegible]　　　　日期：　15 年 1 月12日
